# Rhinos in the Parks: An Island-Wide Survey of the Last Wild Population of the Sumatran Rhinoceros

**DOI:** 10.1371/journal.pone.0136643

**Published:** 2015-09-16

**Authors:** Wulan Pusparini, Paul R. Sievert, Todd K. Fuller, Timothy O. Randhir, Noviar Andayani

**Affiliations:** 1 Department of Environmental Conservation, University of Massachusetts, Amherst, MA, United States of America; 2 Wildlife Conservation Society–Indonesia Program, Tanah Sareal, Bogor, Indonesia; U.S. Geological Survey, UNITED STATES

## Abstract

In the 200 years since the Sumatran rhinoceros was first scientifically described (Fisher 1814), the range of the species has contracted from a broad region in Southeast Asia to three areas on the island of Sumatra and one in Kalimantan, Indonesia. Assessing population and spatial distribution of this very rare species is challenging because of their elusiveness and very low population number. Using an occupancy model with spatial dependency, we assessed the fraction of the total landscape occupied by Sumatran rhinos over a 30,345-km^2^ survey area and the effects of covariates in the areas where they are known to occur. In the Leuser Landscape (surveyed in 2007), the model averaging result of conditional occupancy estimate was $\hat{\psi }\left(SE\left[\hat{\psi }\right]\right)=0.151\left(0.109\right)$ or 2,371.47 km^2^, and the model averaging result of replicated level detection probability $\hat{p}\left(SE\left[\hat{p}\right]\right)=0.252\left(0.267\right)$; in Way Kambas National Park—2008: $\hat{\psi }\left(SE\left[\hat{\psi }\right]\right)=0.468\left(0.165\right)$ or 634.18 km^2^, and $\hat{p}\left(SE\left[\hat{p}\right]\right)=0.138\left(0.571\right)$; and in Bukit Barisan Selatan National Park—2010: $\hat{\psi }\left(SE\left[\hat{\psi }\right]\right)=0.322\left(0.049\right)$ or 819.67 km^2^, and $\hat{p}\left(SE\left[\hat{p}\right]\right)=0.365\left(0.42\right)$. In the Leuser Landscape, rhino occurrence was positively associated with primary dry land forest and rivers, and negatively associated with the presence of a road. In Way Kambas, occurrence was negatively associated with the presence of a road. In Bukit Barisan Selatan, occurrence was negatively associated with presence of primary dryland forest and rivers. Using the probabilities of site occupancy, we developed spatially explicit maps that can be used to outline intensive protection zones for in-situ conservation efforts, and provide a detailed assessment of conserving Sumatran rhinos in the wild. We summarize our core recommendation in four points: consolidate small population, strong protection, determine the percentage of breeding females, and recognize the cost of doing nothing. To reduce the probability of poaching, here we present only the randomized location of site level occupancy in our result while retaining the overall estimation of occupancy for a given area.

## Introduction

The year 2014 marked the bicentennial of the scientific description of the Sumatran rhinoceros (*Dicerorhinus sumatrensis* Fisher 1814), a critically endangered species whose only known wild populations are on the island of Sumatra, Indonesia, and in Kalimantan, Indonesia. The Sumatran rhino is the smallest and most primitive of the five extant species of *Rhinocerotidae*, is most closely related to the woolly rhino (*Coelodonta antiquitatis*) from the Pleistocene ice age [1], and is distantly related to the other Asian rhinoceros species, the Javan rhino (*Rhinoceros sondaicus*) and the Indian rhino (*Rhinoceros unicornis*). Contrary to the implications of its name, the Sumatran rhino once occurred in Myanmar (*D*. *s*. *lasiotis*), Malaysia (Peninsula and Borneo; *D*. *s*. *harrisoni*), and Thailand (D. s. *sumatrensis*, as in Sumatra) [2], but has been extirpated from most of its historical range. The global population of Sumatran rhinos is identified on the International Union for Conservation of Nature (IUCN) red list as Critically Endangered [3], and was estimated to have decreased from 600 animals in 1985 to less than 300 in 1995 [2]. In 2007, the world population was believed to be around 200 individuals [4], and by 2013 it had decreased to less than 100 [5]. The most recent consensus is that the population numbers from 87 to a maximum of 179, with sub-populations ranging in size from 2 to 50 rhinos [6].

Due to its rarity and elusive nature, little is known about the ecology and habitat requirements of the Sumatran rhinoceros. The earliest field studies of rhinos in northern Sumatra occurred in the 1970s and 1980s [7–8], and since then most research on the species has focused on its physiology as it applies to captive breeding [9–14]. In 2008, a systematic assessment of Sumatran rhinos was initiated in Bukit Barisan Selatan National Park, using rhino sign to estimate their distribution and occupancy rate [15]. That work demonstrated that sign surveys were logistically feasible, required minimal equipment (compared to camera trapping), could be completed in a relatively short time period (6–9 months), and could be used for population monitoring purposes.

Assessing the effectiveness of conservation actions depends on our ability to quantify population changes over time [16]. Recently, statistically robust techniques that account for detection probability have been developed for the analysis of occupancy data and are now commonly used in the field of wildlife ecology [17–18]. Our goal was to estimate the occurrence rate of Sumatran rhinos throughout their remaining range on Sumatra, based on island-wide occupancy surveys, and to relate these findings to environmental factors and anthropogenic disturbance. The resulting models of distribution and indices of occurrence can then be employed to evaluate current protection and long-term conservation of the Sumatran rhinoceros.

Identifying the best strategy for conserving the Sumatran rhinoceros is a pressing concern of the international conservation community, and captive breeding, versus protection in the wild, are two approaches being considered [19]. In 1988, the Sumatran Rhino Trust (SRT) developed a captive breeding program, an undertaking that involved four American zoos (Bronx, Cincinnati, Los Angeles, and San Diego), along with the governments of Indonesia and Malaysia, under the American Association of Zoological Parks and Aquarium (AAZPA), and was overseen by the Asian Rhino and Captive Breeding Specialist Group IUCN [20–22]. This program was developed in response to the 1984 declaration by the IUCN, which listed the Sumatran rhino as one of the 12 most endangered species in the world [21]. The philosophy of the SRT is sometimes described as the “Noah’s Ark Paradigm”; i.e., in response to species endangerment a captive breeding program should be developed to ward off extinction [22–23]. Unfortunately, after bringing 40 wild rhinoceroses into captivity and investing $3 million USD [24–25], most of the rhinos died, few offspring were produced, and the program was ultimately deemed unsuccessful [26].

In 1995, Rabinowitz warned that money and effort spent on the capture and breeding of rhinoceroses alone would not solve the problem of declining wild populations. In his essay, he suggested that conservationists would be “helping a species go extinct” if they placed too much emphasis on captive breeding at the expense of "the more difficult job" of protection and management in the field [27]. In support of this perspective, Hutchins and Conway [23] stated that the American Zoo Association (AZA) does not promote captive breeding as a panacea for endangered species.

The Sumatran rhino is now considered to be extinct in Malaysia [28], and the current conservation strategy there is to propagate the remaining rhinos in a captive breeding program [29]. The Borneo Rhino Alliance (BORA) [29] directs this program, and currently it seems there is no chance to re-introduce any rhinos back into the wild [19,26]. On 21 March 2014, a female rhino, named Iman, was captured from the wild in the Danum Valley, Sabah, Borneo, to join Tam (male) and Puntung (female) in the Borneo Rhino Sanctuary, located in the Tabin Wildlife Reserve [30]. These three rhinos are the only representatives left of the Malaysian Borneo Sumatran rhino.

Despite debate regarding the best approach to conserving Sumatran rhinos, most conservationists agree that the primary goal should be to preserve wild animals and their natural habitat [23]. Today, conservationists and wildlife managers may have to consider conservation "triage" for Sumatran rhinos that now exist in the wild as a relatively small metapopulation on the island of Sumatra [31]. On Sumatra, an attempt has been made to conserve the rhinoceros in the wild by guarding them from poachers using Rhino Protection Units. The question remains, is it more feasible to capture all remaining rhinos and save them through the yet-unproven strategy of captive breeding, or is there still a chance for wild population persistence? At this point in time, there is no easy answer.

## Methods

**Fig 1:**
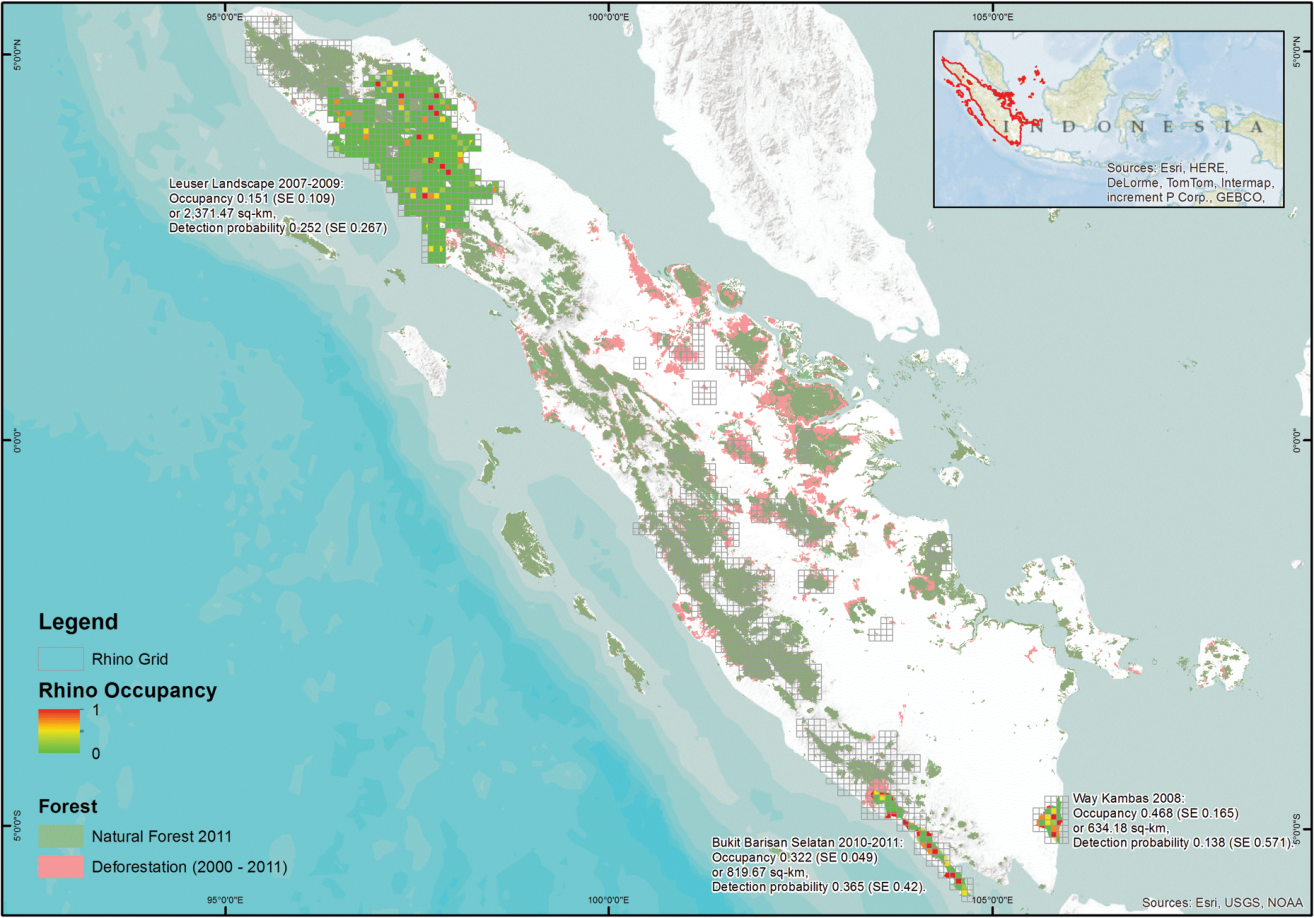
Randomized distributions patter of the last wild population of Sumatran rhinoceros based on occupancy probability.

Permission to conduct this field study was provided by the Director General of Nature Conservation for Gunung Leuser National Park (96° 35’- 98° 30’ East and 2° 50’- 4° 10’ North), Way Kambas National Park (106° 32’- 106° 52’ East and 4° 37’- 5° 15’ South), and Bukit Barisan Selatan National Park (103.3°E 4.51°S-104.7°E 5.93°S) (Fig 1). Since our study only used occurrence sign, and thus no direct interaction with animals, approval from the Institutional Animal Care and Use Committee (IACUC) was not required.

### Study Area

Sumatra is one of more than 17,000 islands in the Indonesian archipelago, and is the largest volcanic island in the world (480,793 km^2^) [32]. Geologically, Sumatra, together with Java and the islands of Nusa Tenggara, is part of the ‘Sunda Archipelago Arc’ formed between 15 and 3 M years BP. Compared to the rest of the Indonesian archipelago, Sumatra is geologically young, and climatic changes and fluctuations of sea level during the Quaternary glacial and inter-glacial periods shaped the present landscape [33]. According to Verstappen (1973) *in* [33], Sumatra Island can be divided into five physiographical units: 1) the coastal strip of the west coast, 2) mountain zone: the Barisan range and the central graben, 3) the eastern piedmont, 4) the well-drained eastern lowlands, and 5) the eastern swampy lowlands and islands to the west and east of Sumatra. The equator passes through the center of Sumatra, dividing it into almost equal-sized northern and southern sections. Temperatures at low elevations are uniformly high, with mean monthly temperatures of 25–27°C. Rainfall is abundant and evenly distributed throughout the year. Sumatra is also divided into five bioclimates/rainfall regimes: subhumid (precipitation 1,000–1,500 mm/year); humid (1,500–2,000 mm/year); very humid (2,000–2,500 mm/year); superhumid (2,500–3,000 mm/year); and hyperhumid (>3,000 mm/year) [33]. Based on these categories, 90% of Sumatra consists of very humid, superhumid and hyperhumid bioclimates.

During 2007–2009, the forest habitat available on Sumatra was surveyed for occurrence of large mammals by eight organizations (Wildlife Conservation Society, Fauna & Flora International, Zoological Society of London, Leuser International Foundation, World Wildlife Fund, Rhino Foundation of Indonesia, Sumatran Tiger Protection and Conservation Program, and Durrell Institute of Conservation and Ecology) [34]. The surveys confirmed the presence of Sumatran rhinoceros in only three areas: Leuser Landscape (LL; 2 May 2007–1 March 2009); Way Kambas NP (WKNP; 6 January– 11 March 2008); and, Bukit Barisan Selatan NP (BBSNP; 19 October 2007–25 June 2008; [15]). For this last study area, we analyzed data collected during the second survey (28 September 2010–2 February 2011) that was conducted by the Wildlife Conservation Society. These three areas effectively contain the last remaining populations of wild Sumatran rhinoceros in the world, now that the populations in Malaysia (Sabah and Peninsular) are extinct [35–36, 19, 28]. The newly discovered population in West Kalimantan is thought to be too small to be viable [37,6].

During 1985–2007, 49.3% of forest was lost in Sumatra and currently only 29% of Sumatra is still forested [38]. The Leuser Landscape is located in northern Sumatra and covers 26,000 km^2^, while BBSNP and WKNP are located in southern Sumatra and cover 3,500 km^2^, and 1,293 km^2^, respectively. The LL is mostly forested and located in the provinces of Aceh and North Sumatra [39]. The BBSNP was 77% forested in 2000 but since then has lost forest cover at a rate of 0.57%/year, primarily due to the establishment of coffee plantations [40]. Historically, before establishment of the park, some areas were designated as logging concessions and 60 km of logging trails exist today [41]. WKNP is 43% forest, most of which has regrown following logging, contains 234 km of logging trails, and is losing forest cover at a rate of 1.01%/year [41]. Both BBSNP and the LL are located on the Barisan geanticline that was formed by Tertiary volcanic activity along the west coast of Sumatra [33]. WKNP is located in the eastern swamp lowlands physiographic unit. Rhino Protection Units have patrolled to reduce poaching of wild Sumatran rhinos in BBSNP since 1995, in WKNP since 1998, and in Gunung Leuser National Park (GLNP; within LL), since 2012.

### Data Collection

Surveys across the island followed the protocol for the Sumatran rhinoceros [42], adapted from the landscape-wide patch occupancy protocol [43, 44]. In summary, a 72.25-km^2^ grid (8.5 x 8.5 km) was defined as the sampling unit since this area is slightly larger than the maximum home range size reported for a Sumatran rhino (60 km^2^; [8]). The number of grids surveyed was 337 in the LL, 55 in BBSNP, and 28 in WKNP. The total length of survey routes was 4,479 km in the LL (mean = 13.29 km/grid, range = 2–38 km/grid), 340 km in WKNP (mean = 12.14 km/grid, range = 1–39 km/grid), and 1,042 km in BBSNP (mean = 18.95 km/grid, range = 4–41 km/grid), for a total of 5,861 km across 421 survey grids. This is by far the most substantial effort spent for assessing the Sumatran rhinoceros.

Surveys were conducted by four teams, each composed of four individuals that searched for rhino sign along paths believed to have a high probability of use by rhinos. Teams recorded signs of rhino presence and any signs of natural or human disturbance. Only footprints were used in analyses of surveys from BBSNP and WKNP to avoid false detection from tapirs (*Tapirus indicus*), and in LL we also used dung and two rare incidents of direct sightings (sound and vision) as signs of presence since tapirs are not found in this area. In addition to the non-random survey routes, a randomly selected 18-km^2^ cell was chosen to ensure randomization of search effort.

### Assessing the Influence of Landscape and Disturbance on Sumatran Rhinos

We evaluated the importance of 17 environmental and anthropogenic covariates on the probability distribution of Sumatran rhinos. Covariates examined included: landcover types, roads, rivers, natural and anthropogenic disturbances, landscape roughness (index of altitude and slope), forest cover, deforestation, and a vegetation index (Normalized Difference Vegetation Index—NDVI) (S1 Table). All covariates for each site were checked for autocorrelation, and only one of the correlated variables was included in a model if the absolute value of Pearson’s correlation coefficient was greater than ±0.70.

Species often respond to environmental and anthropogenic factors at different scales. To assess this, a kernel density estimator was chosen for extracting the value of covariates to represent pattern intensity across a range of spatial scales. We assessed landscape characteristics and disturbance factors (covariates) for different cell sizes and linear distances. For each covariate, cell sizes of 50 m, 100 m and 200 m were chosen as the ‘grain’ sizes that rhinos might respond to. Ten spatial distances (kernel width) from 500 to 5,000 m (in 500-m increments) were created in order to capture various scales of pattern intensity, using the kernel density tool for point and polyline data, and a weighted focal statistic for the polygon and raster data.

Multi-scale analyses were conducted for all of the covariates, except the gradient data from a Digital Elevation Model (DEM) and the vegetation index. To extract the gradient matrix of elevation and the vegetation index, we used the ArcGIS Geomorphometry and Gradient Metrics toolbox [45]. Roughness was one of the surface matrices extracted from DEM, and was composed of overall variability in surface height (non-spatial) and the local variability in slope (spatial) [46]. Curvature of the NDVI summarized the combination of amplitude and spatial characteristics of NDVI local peaks where the index value is at a maximum. We identified the most appropriate scale for our covariates using a single-season occupancy model. The most parsimonious model with the corresponding covariate scale was assumed to be best. Due to the wide variation in environmental characteristics between the three areas, a different subset of the 17 covariates was evaluated for each area (S2 Table).

### Data Analysis

All analyses were conducted using PRESENCE var. 8.3 [47] and R version 2.8.1. We developed a detection matrix of 1s and 0s (detection or non-detection of sign, respectively) using a sampling occasions of 1-km in length. A detection indicates that sign was found, while no detection could result from either true absence or false absence. Using ArcGIS 10.1, the survey track log was downloaded from GPS and overlaid with rhino sign locations. We then calculated the 3D length (using the DEM) of each survey route and subdivided these routes into 1-km detection segments that served as spatial replicates. Grid cells varied with regard to area that was unsuitable for rhinos (e.g., sea or human settlement), resulting in different transect lengths between grids. In creating our detection matrix, we defined a full trial as the longest transect measured across all grids. Shorter transects were made equivalent to a full trial by recording missing values for the difference in transect length between a given transect and the longest transect. Missing values were ignore in the log-likelihood function [48], and therefore did not bias our analyses.

To estimate occurrence, we used site-occupancy models [49–52], also known as zero-inflated binomial models [53], with an underlying Markov models for spatial dependence [54]. A site-occupancy model is a hierarchical logistic regression that uses a logit link function and employs Bernoullian random effects to model the probability of observation (detection) and process (occupancy). This modeling approach was developed specifically to estimate the proportion of an area occupied by a species, but it can also be used to predict the geographical range and habitat characteristics of a species [51]. Estimation of detection probabilities and occurrences was conducted by incorporating occupancy probabilities directly into the model likelihood [49–52]. Occupancy with spatial dependence decomposed the detection process into rhino presence at a segment, and rhino detection given presence on a segment is modeled as a first-order spatial Markov process [54]. An occupancy model is essentially a discrete random-effects model that includes variable values assumed to be drawn from a Bernoulli or Poisson distribution which correspond to the true, but imperfectly observed, state of occurrence [55]. The model was run using a 1-km segment length in a cluster sampling, spatial replication scenario. In order to avoid multi-collinearity issues, we examined correlations between covariates and if |r| was > 0.70 they were not used in the same model. We modified the basic approach used by Karanth et al. [56] in a two-stage process by modeling covariates affecting the detection and ecological process. Instead of first using a global model of occupancy to correct the covariates of detection probability, we used constant covariates of detection and combined various ranges of covariates to get the ‘best’ set for predicting occupancy (S3, S5 and S7 Tables). The second stage of our modeling used the covariates of occupancy from the first stage, and tested different combinations of covariates in the detection probability (S4, S6, and S8 Tables). We assumed no covariates in modeling the Markovian dependence. All modeling was conducted using PRESENCE single season with correlated detection [54], and the *psi* estimates output was further calculated to take into account the effect of the correlated detection (R code provided by James Hines, Arnaud Lyet personal comm.). The model averaged *Psi* and associated SE were further weighted based on the available areas within each sampling grid that could be habitable [56]; i.e., a sampling grid with 100% coverage of forest received a weight of 1 and coverage less than that, a lesser weight. Site-level-occupancy patterns for each area were further randomized to obscure the exact location of the area and reduce the possibility of poaching. A map showing the true occurrence pattern is available for conservation purposes only and can be requested from the authors.

## Results

### Multiscale Assessment of Covariates

Each of the covariates, with associated scale, listed in S1 Table were run in single season occupancy—constant detection models. Several of the covariates were correlated with rhino occurrence (failure to converge defined as non-fit for the respective dataset), but often at a spatial scale that differed between study areas (S2 Table). For example, road presence was significant at the ‘grain’ of 100 m for all three areas, but at a kernel radius of 4,000 m in LL, 5,000 m in WKNP, and 1,500 m in BBSNP. Thus, road presence was an important covariate, but it affected rhino occurrence at a shorter distance (1.5-km from the road) in BBSNP compared to WKNP (5-km). The set of covariates for each area was tested for collinearity and none exceeded the chosen threshold of |r|>0.7 (S1, S2, and S3 Figs).

### Leuser Landscape (LL)

The first stage of modeling was to assess covariates affecting occupancy probability with constant detection (S3 Table, all the models with ΔAIC < 7). The most parsimonious model showed that occupancy with constant detections was mainly affected by Primary Dry Land Forest, River, and Road. In the next step we focused these covariates on occupancy and tried to fit a range of site covariates with detection probability (S4 Table). Our results showed that curvature of NDVI and Roughness were affecting detection. This model of *ψ(PDF + River + Road)*,*θ(*.*)*,*θ'(*.*)*,*p(NDVI + Roughness)* had a 52% chance of being the best model based on AIC weight. Primary dry land forest had positive association with occupancy (in logit scale: 6.09(3.88), S4 Fig), as did river (8.67(4.94), S5 Fig), while presence of road had a negative association (however, the value is very small and approaching zero; Table 1A).

**Table 1:** Untransformed parameter estimates.

**A. Leuser Landscape**									
**Model**	**psi(Int) (SE)**	**psi(PDF) (SE)**		**psi(River) (SE)**			**psi(Road) (SE)**		
ψ(PDF + River + Road),θ(.),θ'(.),p(NDVI + Roughness)	-5.95 (2.62)	6.09 (3.88)		8.67 (4.94)			-22066443.23 (2.62437E+11)		
ψ(PDF + River + Road),θ(.),θ'(.),p(River)	4.88 (8.40)	19.92 (22.27)		-11.78 (15.14)			-17945717.42 (15512506743.60)		
ψ(PDF + River + Road),θ(.),θ'(.),p(River + Disturbance)	5.09 (7.97)	20.43 (23.09)		-12.15 (14.46)			-27599545.41 (1945171990129.93)		
ψ(PDF + River + Road),θ(.),θ'(.),p(NDVI)	-5.86 (2.25)	4.89 (2.82)		7.35 (3.89)			-22963173.05 (497366108644.48)		
ψ(PDF + River + Road),θ(.),θ'(.),p(.)	-6.43 (2.16)	4.75 (2.56)		7.38 (3.61)			-20901005.39 (211520464377.18)		
**B. Way Kambas National Park**									
**Model**					**psi(Int) (SE)**			**psi(Road) (SE)**	
ψ(Road),θ(.),θ'(.),p(.)					1.49 (1.86)			-33.33 (22.53)	
ψ(Road),θ(.),θ'(.),p(Forest)					2.17 (2.83)			-31.01 (27.97)	
ψ(Road),θ(.),θ'(.),p(Disturbance)					1.57 (1.87)			-33.59 (22.76)	
ψ(Road),θ(.),θ'(.),p(NDVI)					1.53 (1.78)			-33.64 (22.72)	
ψ(Road),θ(.),θ'(.),p(River)					1.49 (1.86)			-33.26 (23.07)	
**C. Bukit Barisan Selatan National Park**									
**Model**			**psi(Int) (SE)**			**psi(PDF) (SE)**			**psi(River) (SE)**
ψ(PDF + River),θ(.),θ'(.),p(DLA)			4.42 (2.24)			-6482.01 (31565305.04)			-9.16 (4.46)
ψ(PDF + River),θ(.),θ'(.),p(DLA + Disturbance)			4.34 (2.21)			-6848.94 (70730180.01)			-9.07 (4.41)
ψ(PDF + River),θ(.),θ'(.),p(DLA + Disturbance + Forest)			4.17 (2.13)			-21003.10 (10)			-8.72 (4.25)
ψ(PDF + River),θ(.),θ'(.),p(Forest)			4.32 (2.62)			-7367.17 (95562737.74)			-8.48 (4.79)
ψ(PDF + River),θ(.),θ'(.),p(.)			4.55 (3.11)			-7724.96 (131835924.27)			-8.91 (5.51)
ψ(PDF + River),θ(.),θ'(.),p(Disturbance + Forest)			3.48 (2.14)			-19465.39 (10)			-7.33 (4.20)
ψ(PDF + River),θ(.),θ'(.),p(Disturbance)			3.96 (2.56)			-53893.62 (10)			-8.07 (4.74)

PDF, Primary Dryland Forest; DLA, Dryland Agriculture.

The total fraction of area for the model averaged occupancy probability is $\hat{\psi }\left(SE\left[\hat{\psi }\right]\right)=0.151\left(0.109\right)$ with detection probability $\hat{p}\left(SE\left[\hat{p}\right]\right)=0.252\left(0.267\right)$. This translated into the area occupied by Sumatran rhinos in 2007 being 2,371.47 km^2^ out of 15,751.24 km^2^ of total surveyed area. The randomized site-specific probability of occupancy is shown in Fig 1, with red indicating probability of occupancy of 100% and green indicating 0%.

### Way Kambas National Park (WKNP)

Following the same procedure, the occupancy model with correlated detection showed that roads affected the occupancy probability (S5 Table), and none of the site covariates affected the detection probability (S6 Table). The model of *ψ(Road)*,*θ(*.*)*,*θ'(*.*)*,*p(*.*)* had a 51% chance of being the most parsimonious model among all, as shown by the adjusted AICc for small sample size [57]. Roads showed a negative relationship with the occurrence of Sumatran rhinos in Way Kambas National Park (in logit scale: -33.33(22.53), Table 1B, S6 Fig). The total fraction of area for the model averaged occupancy probability is $\hat{\psi }\left(SE\left[\hat{\psi }\right]\right)=0.252\left(0.267\right)$ with detection probability $\hat{p}\left(SE\left[\hat{p}\right]\right)=0.138\left(0.571\right)$. This translated into area occupied by Sumatran rhinoceros in 2008 as 634.18 km^2^ out of 1,355.21 km^2^ total surveyed area. The randomized site-specific probability of occupancy is shown in Fig 1.

### Bukit Barisan Selatan National Park (BBSNP)

Primary dryland forest and river were the covariates shown to affect the occupancy probability with constant detection probability (S7 Table). The second stage of modeling showed that the combination of these covariates with Dryland agriculture as site covariates for detection probability, and *ψ(PDF + River)*,*θ(*.*)*,*θ'(*.*)*,*p(DLA)* was the most parsimonious model with a 54% chance of being the best model (S8 Table) based on adjusted AICc for small sample size. River had a negative relationship with the probability of occupancy (in logit scale: -9.16(4.46), S7 Fig), and primary dry land forest also had a negative relationship (however the value was very small and approached zero; Table 1C). The total fraction of area for the model averaged occupancy probability is $\hat{\psi }\left(SE\left[\hat{\psi }\right]\right)=0.322\left(0.049\right)$ with detection probability $\hat{p}\left(SE\left[\hat{p}\right]\right)=0.365\left(0.42\right)$. This translated into an area occupied by Sumatran rhinoceros in 2010 of 819.67 km^2^ out of 2,545.18 km^2^ surveyed (Fig 1).

## Discussion

Current knowledge of the ecology of Sumatran rhinos in the wild is largely based on field studies in the 1970s [7] and 1980s [8]. Our analysis provides a current quantitative assessment of Sumatran rhinoceros occurrence and updates the 1986 rhino distribution findings [8] for the Leuser region. Although these ‘current’ data were collected several years ago, they are still the best and most up to date assessment for this critically endangered species.

Prior to our study, no systematic surveys had been conducted to estimate the distribution of rhinos throughout Sumatra. Using a hierarchical modeling approach, we were able to assess the distribution of Sumatran rhinos while taking into account the low probability of detecting signs in the wild. This approach was first tested in Bukit Barisan Selatan National Park in 2008 [15]. Errors of commission, when a species is predicted to be present but is not observed, reduce the accuracy of predictions from wildlife-habitat models [58]. In our analyses, we used detection/non-detection data to estimate spatially explicit occurrences, which were then used to develop rhino distribution maps for the island of Sumatra.

### Leuser Landscape

In the Leuser landscape, we found that Sumatran rhinos are primarily concentrated in the central region. Comparing this occurrence map to the International Union for Conservation of Nature’s distribution map [3] and van Strien’s map [8], it appears that in addition to the ‘core’ population, there may be several small populations outside the national park, and thus receive no institutional protection. We estimated the size of the area rhinos occupied by the rhinos in the LL to be 2,371 km^2^, a relatively small area given the size of the landscape (26,000 km^2^), with total occurrence of 0.151 (0.109).

In Leuser, the presence of primary dry land forest and river was important for Sumatran rhino, and the presence of roads had a negative impact. Contrary to anecdotal suggestions that the optimum locations for rhinos are in lowlands, our model showed that topographic roughness did not influence rhino occurrence. Sumatran rhinos were found both at low elevation sites with less rugged terrain, and in high elevation habitats.

Study in 1985 used footprint size to identify individual rhinos and estimated a density of 10 rhinos/100 km^2^ in Leuser, or a total of 130–200 animals in the area [8]. The current population is likely lower than this estimate, but successful breeding still occurs, as indicated by a 2014 video trap set by the Leuser International Foundation. We suggest the next step for saving the LL rhino population is to secure the core area while at the same time trying to assess the small isolated populations scattered outside the core area. Given the current landscape, it is unlikely that these small populations are connected, but management plans should attempt to consolidate them into a single large population. Because rhino occurrence is negatively influenced by roads, we suggest that any area proposed for consolidating the population be located far from roads.

### Way Kambas National Park

Of the three parks currently supporting Sumatran rhinos, WKNP is by far the smallest and contains the most unique habitats. Unlike the other two national parks, this 1,293-km^2^ park is dominated by swamp forest, along with secondary lowland forest that resulted from intensive logging in the 1960s and 1970s. Following logging in the area, rhinos were believed to be locally extinct [7], but in the 1990s they were ‘rediscovered’ [59]. The park is located in southeast Sumatra and is bordered on the east by the sea, while its remaining borders are in direct contact with the surrounding settlements [59]. It encloses the Sumatran Rhino Sanctuary (SRS), a semi-captive breeding facility for the Sumatran Rhinoceros. In WKNP, Sumatran rhino occurrence is negatively associated with roads, but disturbance was unrelated to rhino occurrence. Perhaps roads, which represent constant human presence, do not disturb the rhinoceros as much as occasional human presence in the park.

Despite being the smallest of the three areas, we estimate that WKNP has a rhino occurrence probability of 0.468(0.165). Thus, it appears that WKNP has the highest occurrence rate of rhinos in Sumatra. Rhinos occupied 634 km^2^ of the park area, similar to the occurrence area in BBSNP. The last known poaching incident in the park was in 2007, and no incident of reported rhino poaching ever since, though there are signs of elephant killing inside the park. The small size of the park should allow for rapid inspection by protection teams. Because the occurrence rate of rhinos is high in WKNP, it is critical that their population be protected, even though its size is likely to remain small due to the limited size of the park. Rhino calves were recently born both in the Sanctuary and in the wild. The wild-born calf was photographed with its mother by a rhino protection unit in 2010 and estimated to be 10 months old.

### Bukit Barisan Selatan National Park

Of the three parks, BBSNP is the most studied area. Comparing the overall rhino occupancy in 2008 (0.32; 0.09) with the current estimate (0.322; 0.049), the distribution seems stable with an area occupied in 2010–2011 of ~820 km^2^. Interestingly, the same two covariates that were significant for LL also affected occurrence of rhino in BBS, but in the opposite way. Sumatran rhinos in BBS were found in areas with fewer rivers (mostly along ridges), and appeared to avoid primary dryland forest (though the effect was small; Table 1C).

BBSNP has long been thought to be the area with highest abundance of Sumatran rhinos in Sumatra [4, 60]. During 1998–2005, rhino sign was frequently found in the park, with most rhino activity occurring in the central region [61]. In 2004–2005, the national road crossing the central region was upgraded and currently it receives heavy use since it connects the eastern part of the province with the large west coast city of Bengkulu. WCS has suggested establishing a non-violated zone in the middle of the park [62], known as an Intensive Protection Zone (IPZ), based on conservation approaches used to conserve African rhino species.

Our observations indicated that there may be two rhino sub-populations outside of the proposed IPZ. The persistence of these sub-populations has not been confirmed since subsequent surveys (2012 –WCS unpublished, and 2013 –WWF and YABI unpublished) were restricted to the IPZ area. However, data from current camera trapping and fecal DNA surveys (unpublished data) indicate that the population likely has crashed, and suggests that the remaining population might not be large enough to survive on its own. One possibility is to consolidate individuals in a new area with very strong protection.

## Conclusions

Currently, there is decreasing support in the conservation community to maintain Sumatran rhinos in their natural habitat [19], and increasing pressure to remove all remaining Sumatran rhinos from the wild with the hope of developing a successful captive breeding program for the species. Pessimism regarding maintenance of a wild rhino population on Sumatra is understandable since the island has a population of 50 million people [32] and an annual population growth rate of 2.4% (mean from 10 provinces, census 2010 [32]). As a result, there is continuing pressure to develop natural habitats, as reflected by the national government's proposal of an economic corridor in which Sumatra would be the center for production of natural resources and the nation’s energy reserve [63]. This plan is likely to have significant negative impact on rhinoceroses since the development of roads would give poachers increased access [64–65]. In addition, the proposed developments would reduce the area of old-growth forest on Sumatra, a habitat that declined by 40% between 1990 and 2010 [66].

With uncertainty surrounding the actual rhino population size, it is better to assume a small population and apply appropriate management accordingly. The question that still needs to be answered is: how small is it? Furthermore, the relationship between a species distribution and its population size is not monotonic, especially for a small population of large mammals. A single individual, in search of food, security, and/or breeding partner, can move over vast areas. Even without anthropogenic pressure on the population, the combined impacts of demographic and environmental stochasticity can lead to high rates of extinction in growing populations that are fewer than 15, and in non-growing populations of fewer than 40 [6]. Conservation step therefore should be taken immediately without waiting for hard to get information in population sizes.

However, by removing every rhino from the Sumatran landscape, we fear that their natural landscape would be at a greater risk of development. The presence of Sumatran rhinos can provide a strong argument for protecting vast landscapes, as the mountain gorilla (*Gorilla beringei beringei*) has for Africa. Protected habitat is necessary for sustaining a viable rhino population, and for providing a place for animals that might be produced in a successful captive breeding program. We believe it is not too late to conserve Sumatran rhinos through protection of their wild populations. Some sub-populations may be too small to survive on their own (e.g., BBSNP and some in LL), and for these it is vital to consolidate them into a single protected population.

In summary, our four recommendations going forward are to:

1. Consolidate the small population in BBSNP and the outside of the core population of LL. This will require strong political will and major financial support.
2. Give strong protection for the remaining core populations in LL and WKNP by using our maps (Un-randomized maps, available upon request for strict conservation purposes) to better identify an Intensive Protection Zone (IPZ).
3. Determine the percentage of breeding females in each population and each population’s genetic variability. We recognize that DNA from rhino feces degrades quickly in tropical rainforests and that dung from such a small population individual is hard to find, but new developments in environmental genetics (E-DNA) could be applied, even in the scattered small populations outside the core to confirm rhino presence.
4. Recognize that the result of doing nothing is likely to be extinction of the Sumatran rhino, as occurred with the Javan Rhino in Vietnam [67].

The Indonesian Government is positioned to be the leader in Sumatran rhino conservation, and should put forth the effort to save this iconic species.

## Supporting Information

S1 Fig Pearson’s Correlation Matrix for Selected Covariates—Leuser Landscape. (TIFF)

S2 Fig Pearson’s Correlation matrix for Selected Covariates—Way Kambas National Park. (TIFF)

S3 Fig Pearson’s Correlation Matrix for Selected Covariates—Bukit Barisan Selatan National Park. (TIFF)

S4 Fig Occurrence Probability and Primary Dry Land Forest, Leuser Landscape 2007–2009. (TIFF)

S5 Fig Occurrence Probability and River, Leuser Landscape 2007–2009. (TIFF)

S6 Fig Occurrence Probability and Major Road, Way Kambas NP 2008. (TIFF)

S7 Fig Occurrence probability and River Bukit Barisan Selatan NP 2010–2011. (TIFF)

S1 Table List of covariates. (DOCX)

S2 Table Different scale of covariates use in each area. (DOCX)

S3 Table Leuser Landscape– 2007–2009. Model selection results; roles of covariates in determining probability of occupancy Sumatran rhino, with constant detection probability *p* on 1km long replicates, using the Hines et al. (2010) model. Number of sites = 337. Covariates considered Primary Dryland Forest (PDF), River, Road Density (Road), Curvature of NDVI (NDVI), Roughness, and Secondary Dryland Forest (SDF).
(DOCX)

S4 Table Leuser Landscape– 2007–2009. Model selection results; roles of covariates in Sumatran rhinoceros occupancy in Leuser Landscape, based on modeling probability of detecting rhino sign *p* on 1km long replicates using the Hines et al. (2010) model. Number of sites = 337. Covariates considered Primary Dryland Forest (PDF), River, Road Density (Road), Curvature of NDVI (NDVI), Roughness, and Disturbance.
(DOCX)

S5 Table Way Kambas NP—2008. Model selection results; roles of covariates in determining probability of occupancy Sumatran rhino, with constant detection probability *p* on 1km long replicates, using the Hines et al. (2010) model. Number of sites = 28. Covariates considered Road Density (Road), Forest, Deforestation, and Curvature of NDVI (NDVI).
(DOCX)

S6 Table Way Kambas NP– 2008. Model selection results; roles of covariates in Sumatran rhinoceros occupancy in Leuser Landscape, based on modeling probability of detecting rhino sign *p* on 1km long replicates using the Hines et al. (2010) model. Number of sites = 28. Covariates considered Road Density (Road), Forest, Disturbance, Curvature of NDVI (NDVI), and River.
(DOCX)

S7 Table Bukit Barisan Selatan NP– 2010–2011. Model selection results; roles of covariates in determining probability of occupancy Sumatran rhino, with constant detection probability *p* on 1km long replicates, using the Hines et al. (2010) model. Number of sites = 55. Covariates considered Primary Dryland Forest (PDF), River, Secondary Dryland Forest (SDF), Deforestation, and Disturbance.
(DOCX)

S8 Table Bukit Barisan Selatan NP– 2010–2011. Model selection results; roles of covariates in Sumatran rhinoceros occupancy in Leuser Landscape, based on modeling probability of detecting rhino sign *p* on 1km long replicates using the Hines et al. (2010) model. Number of sites = 55. Covariates considered Primary Dryland Forest (PDF), River, Dryland Agriculture (DLA), Disturbance, and Forest.
(DOCX)
