# Correction: Rhinos in the Parks: An Island-Wide Survey of the Last Wild Population of the Sumatran Rhinoceros

**DOI:** 10.1371/journal.pone.0139982

**Published:** 2015-10-05

**Authors:** Wulan Pusparini, Paul R. Sievert, Todd K. Fuller, Timothy O. Randhir, Noviar Andayani

## Notice of Republication

This article was republished on September 22, 2015, due to the release of confidential or copyrighted material. Please download this article again to view the correct version.
