# Supplementary figures and images for: Rhinos in the Parks: An Island-Wide Survey of the Last Wild Population of the Sumatran Rhinoceros
Source: PLoS One. 2015 Sep 16;10(9):e0136643. doi: 10.1371/journal.pone.0136643 (PMC4574046; doi:10.1371/journal.pone.0136643)

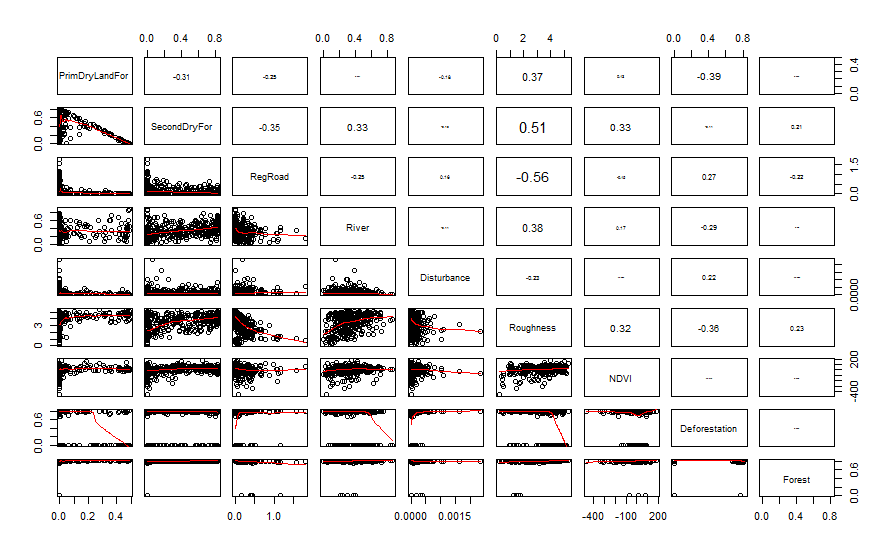

Supplement: S1 Fig — (TIFF) [file pone.0136643.s001.tiff]

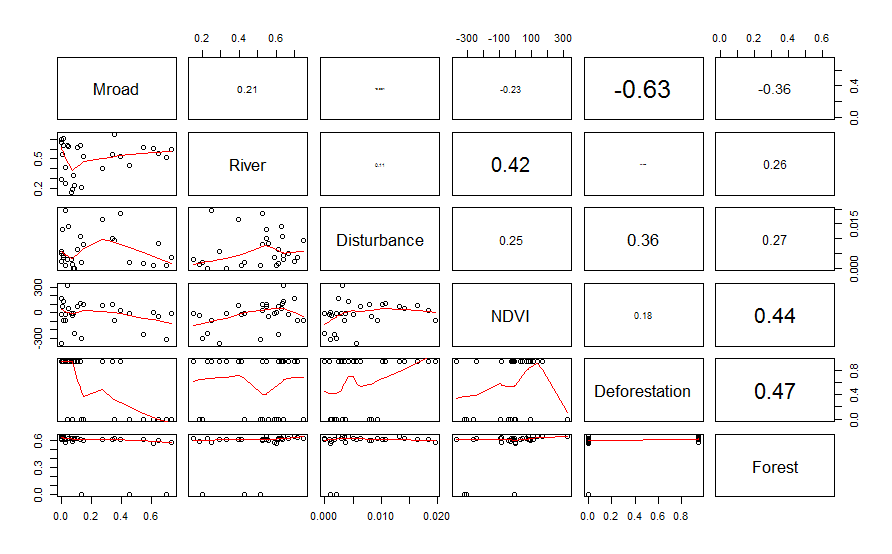

Supplement: S2 Fig — (TIFF) [file pone.0136643.s002.tiff]

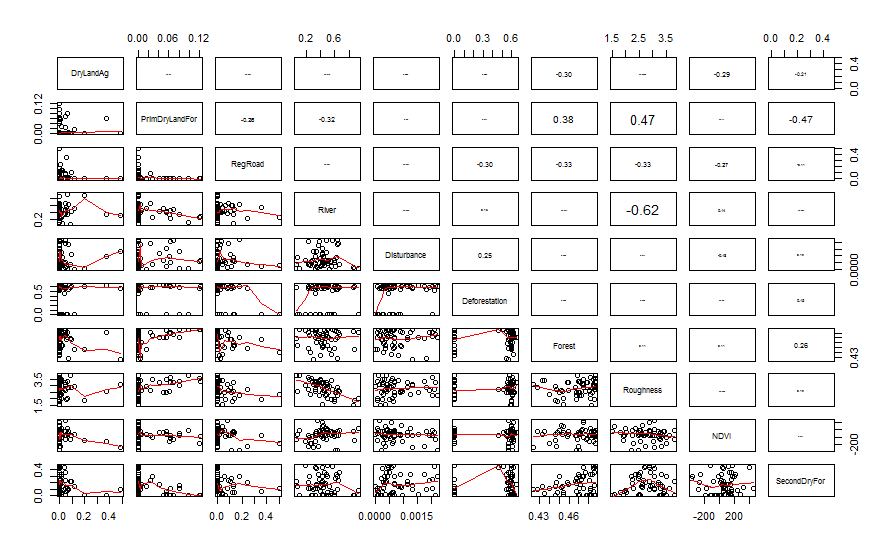

Supplement: S3 Fig — (TIFF) [file pone.0136643.s003.tiff]

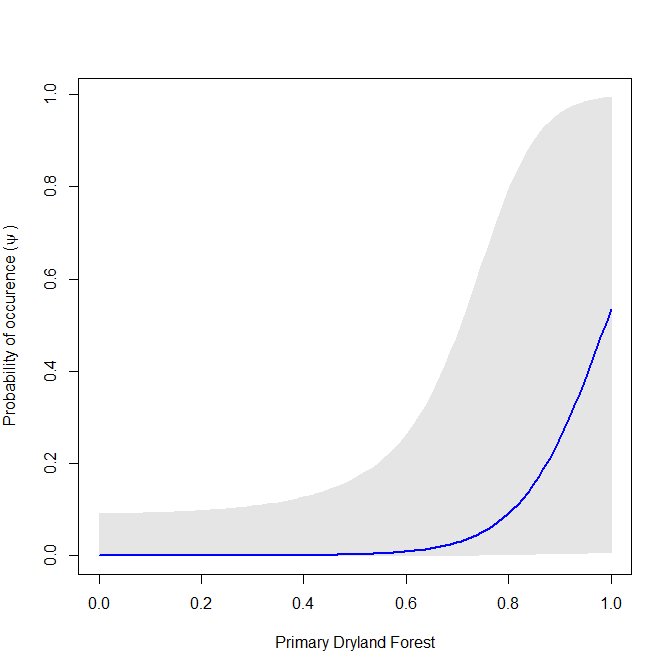

Supplement: S4 Fig — (TIFF) [file pone.0136643.s004.tiff]

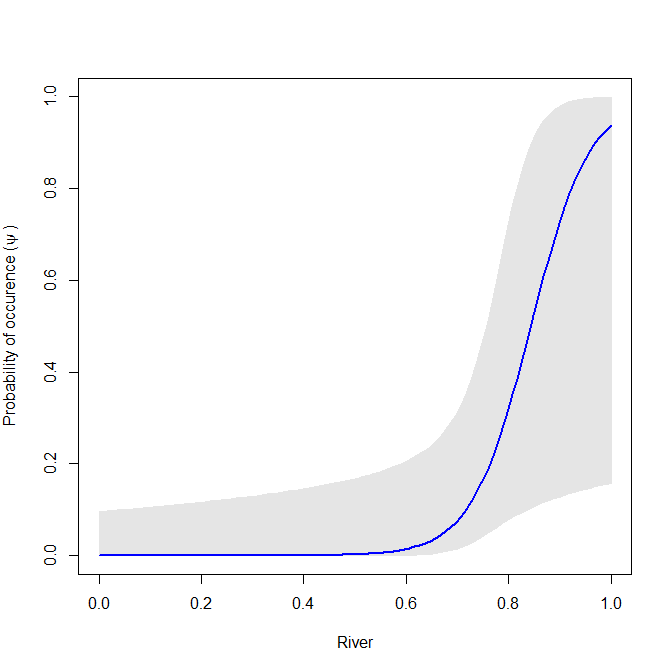

Supplement: S5 Fig — (TIFF) [file pone.0136643.s005.tiff]

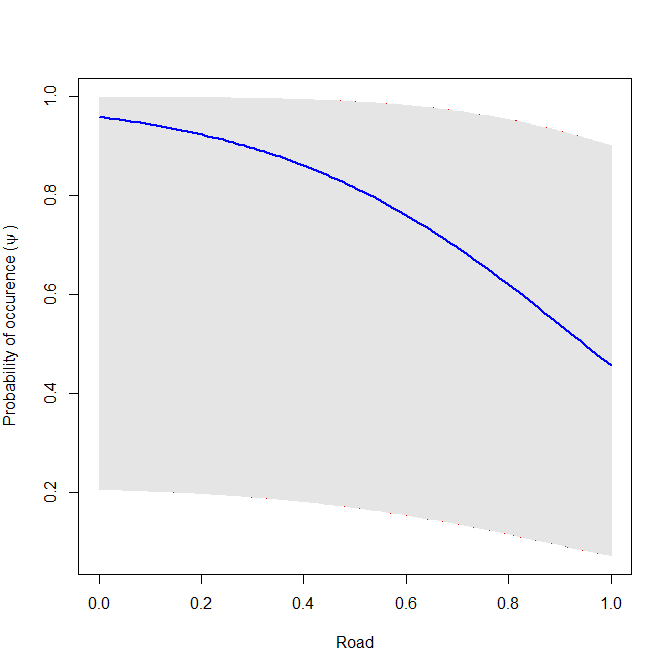

Supplement: S6 Fig — (TIFF) [file pone.0136643.s006.tiff]

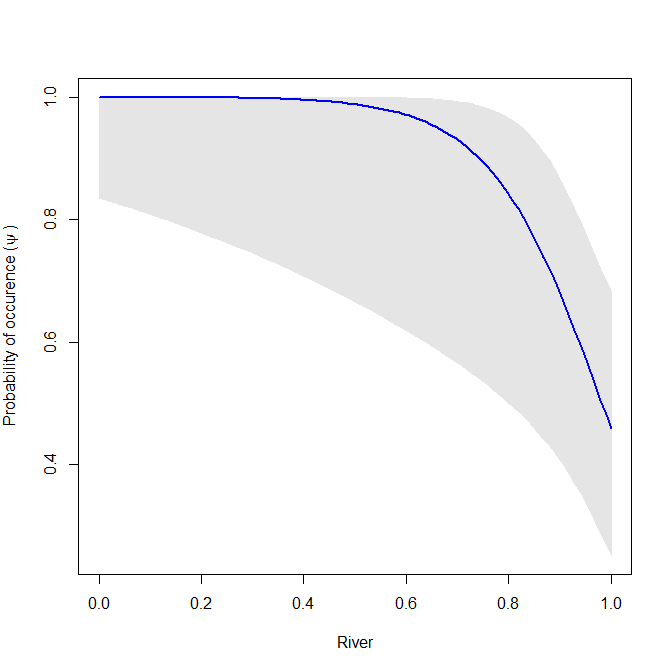

Supplement: S7 Fig — (TIFF) [file pone.0136643.s007.tiff]
