# Supplementary material for: Rhinos in the Parks: An Island-Wide Survey of the Last Wild Population of the Sumatran Rhinoceros
Source: PLoS One. 2015 Sep 16;10(9):e0136643. doi: 10.1371/journal.pone.0136643 (PMC4574046; doi:10.1371/journal.pone.0136643)
Supplement: S1 Table — (DOCX) [file pone.0136643.s008.docx]

### S1 Table. List of available covariates.

| No | Covariate – Class | Type | Year | | Source |
| --- | --- | --- | --- | --- | --- |
|  | ***Landcover*** |  |  |  | |
| 1 | Landcover Type – Brush | Patch | 2003 | Indonesian Ministry of Forestry – BAPLAN | |
| 2 | Landcover Type – Dry Land Agriculture | Patch | 2003 | Indonesian Ministry of Forestry – BAPLAN | |
| 3 | Landcover Type – Dry Land Agriculture with Brush | Patch | 2003 | Indonesian Ministry of Forestry – BAPLAN | |
| 4 | Landcover Type – Primary Dry Land Forest | Patch | 2003 | Indonesian Ministry of Forestry – BAPLAN | |
| 5 | Landcover Type – Secondary Dry Land Forest | Patch | 2003 | Indonesian Ministry of Forestry – BAPLAN | |
| 6 | Landcover Type – Savanna | Patch | 2003 | Indonesian Ministry of Forestry – BAPLAN | |
| 7 | Landcover Type – Secondary Swamp Forest | Patch | 2003 | Indonesian Ministry of Forestry – BAPLAN | |
| 8 | Landcover Type – Settlements | Patch | 2003 | Indonesian Ministry of Forestry – BAPLAN | |
| 9 | Landcover Type – Swamp Brush | Patch | 2003 | Indonesian Ministry of Forestry – BAPLAN | |
| 10 | Major Road | Line |  |  | |
| 11 | Regular Road | Line |  |  | |
| 12 | River |  | ? | Indonesian Ministry of Forestry | |
| 13 | ***Disturbance – Type*** |  |  |  | |
|  | Natural – Forest Gap | Point | 2007, 2008, 2010 | Field Survey | |
|  | Natural – Landslide | Point | 2007, 2008, 2010 | Field Survey | |
|  | Natural – Flood | Point | 2007, 2008, 2010 | Field Survey | |
|  | Anthropogenic – Fire | Point | 2007, 2008, 2010 | Field Survey | |
|  | Anthropogenic – Logging | Point | 2007, 2008, 2010 | Field Survey | |
|  | Anthropogenic – Forest Clearance | Point | 2007, 2008, 2010 | Field Survey | |
|  | Anthropogenic – Non Timber Forest Product | Point | 2007, 2008, 2010 | Field Survey | |
|  | Anthropogenic – Human presence | Point | 2007, 2008, 2010 | Field Survey | |
|  | Anthropogenic – Poaching | Point | 2007, 2008, 2010 | Field Survey | |
|  | Anthropogenic – Coffee field | Point | 2007, 2008, 2011 | Field Survey | |
|  | Other | Point | 2007, 2008, 2010 | Field Survey | |
| 14 | Roughness | DEM | 2008 | The CGIAR Consortium for Spatial Information | |
| 15 | Forest Cover left 1990-2000 | Patch | 2000 | Gaveau et al. 2007 | |
| 16 | Deforestation 1990-2000 | Patch | 2000 | Gaveau et al. 2007 | |
| 17 | NDVI Curvature | NDVI | 2007, 2008, 2010, & 2012 | MODIS Atmosphere | |
