# Supplementary material for: Rhinos in the Parks: An Island-Wide Survey of the Last Wild Population of the Sumatran Rhinoceros
Source: PLoS One. 2015 Sep 16;10(9):e0136643. doi: 10.1371/journal.pone.0136643 (PMC4574046; doi:10.1371/journal.pone.0136643)
Supplement: S2 Table — (DOCX) [file pone.0136643.s009.docx]

S2 Table. Different scale of covariates use in each area.

| **Leuser Landscape** | | |
| --- | --- | --- |
| Covariates | Cell size (m) | Search radius (m) |
| Primary Dryland Forest | 200 | 4000 |
| Secondary Dryland Forest | 100 | 1000 |
| Road | 100 | 4000 |
| River | 100 | 5000 |
| Disturbance | 200 | 5000 |
| Deforestation | 100 | 1000 |
| Forest | 50 | 500 |
| **Way Kambas** | | |
| Covariates | Cell size (m) | Search radius (m) |
| Road | 100 | 5000 |
| River | 100 | 5000 |
| Disturbance | 50 | 1500 |
| Deforestation | 100 | 500 |
| Forest | 100 | 1500 |
| **Bukit Barisan Selatan** | | |
| Covariates | Cell size (m) | Search radius (m) |
| Dryland Agriculture | 100 | 500 |
| Primary Dryland Forest | 100 | 4500 |
| Secondary Dryland Forest | 200 | 4000 |
| Road | 100 | 1500 |
| River | 100 | 500 |
| Disturbance | 200 | 4500 |
| Deforestation | 100 | 1500 |
| Forest | 50 | 1000 |
