# Supplementary material for: Rhinos in the Parks: An Island-Wide Survey of the Last Wild Population of the Sumatran Rhinoceros
Source: PLoS One. 2015 Sep 16;10(9):e0136643. doi: 10.1371/journal.pone.0136643 (PMC4574046; doi:10.1371/journal.pone.0136643)
Supplement: S3 Table — Model selection results; roles of covariates in determining probability of occupancy Sumatran rhino, with constant detection probability p on 1km long replicates, using the Hines et al. (2010) model. Number of sites = 337. Covariates considered Primary Dryland Forest (PDF), River, Road Density (Road), Curvature of NDVI (NDVI), Roughness, and Secondary Dryland Forest (SDF). (DOCX) [file pone.0136643.s010.docx]

### S3 Table. Leuser Landscape – 2007-2009. Model selection results; roles of covariates in determining probability of occupancy Sumatran rhino*,* with constant detection probability *p* on 1km long replicates, using the Hines et al. (2010) model. Number of sites = 337. Covariates considered Primary Dryland Forest (PDF), River, Road Density (Road), Curvature of NDVI (NDVI), Roughness, and Secondary Dryland Forest (SDF).

| Model | Number of parameters | n | AIC | ΔAIC | AIC weight | Cumulative Weight | Model Likelihood |
| --- | --- | --- | --- | --- | --- | --- | --- |
| ψ(PDF + River + Road),θ(.),θ'(.),*p*(.) | 7 | 337 | 121.15 | 0.00 | 0.53 | 0.53 | 1.00 |
| ψ(PDF + Road + NDVI),θ(.),θ'(.),*p*(.) | 7 | 337 | 123.13 | 1.98 | 0.20 | 0.73 | 0.37 |
| ψ(River + Roughness + Road),θ(.),θ'(.),*p*(.) | 7 | 337 | 124.84 | 3.69 | 0.08 | 0.81 | 0.16 |
| ψ(PDF + Road),θ(.),θ'(.),*p*(.) | 6 | 337 | 125.21 | 4.06 | 0.07 | 0.88 | 0.13 |
| ψ(PDF + Road + SDF),θ(.),θ'(.),*p*(.) | 7 | 337 | 125.64 | 4.49 | 0.06 | 0.94 | 0.11 |
| ψ(PDF + River),θ(.),θ'(.),*p*(.) | 6 | 337 | 127.41 | 6.26 | 0.02 | 0.96 | 0.04 |
| ψ(PDF + River + NDVI),θ(.),θ'(.),*p*(.) | 7 | 337 | 127.69 | 6.54 | 0.02 | 0.98 | 0.04 |
| ψ(PDF + River + Roughness),θ(.),θ'(.),*p*(.) | 7 | 337 | 127.87 | 6.72 | 0.02 | 1.00 | 0.03 |
