# Supplementary material for: Rhinos in the Parks: An Island-Wide Survey of the Last Wild Population of the Sumatran Rhinoceros
Source: PLoS One. 2015 Sep 16;10(9):e0136643. doi: 10.1371/journal.pone.0136643 (PMC4574046; doi:10.1371/journal.pone.0136643)
Supplement: S4 Table — Model selection results; roles of covariates in Sumatran rhinoceros occupancy in Leuser Landscape, based on modeling probability of detecting rhino sign p on 1km long replicates using the Hines et al. (2010) model. Number of sites = 337. Covariates considered Primary Dryland Forest (PDF), River, Road Density (Road), Curvature of NDVI (NDVI), Roughness, and Disturbance. (DOCX) [file pone.0136643.s011.docx]

### S4 Table. Leuser Landscape – 2007-2009. Model selection results; roles of covariates in Sumatran rhinoceros occupancy in Leuser Landscape, based on modeling probability of detecting rhino sign *p* on 1km long replicates using the Hines et al. (2010) model. Number of sites = 337. Covariates considered Primary Dryland Forest (PDF), River, Road Density (Road), Curvature of NDVI (NDVI), Roughness, and Disturbance.

| Model | Number of parameters | n | AIC | ΔAIC | AIC weight | Cumulative Weight | Model Likelihood | Cond Psi total average by area |
| --- | --- | --- | --- | --- | --- | --- | --- | --- |
| ψ(PDF + River + Road),θ(.),θ'(.),*p*(NDVI + Roughness) | 9 | 337 | 115.23 | 0.00 | 0.52 | 0.52 | 1.00 | 0.106 |
| ψ(PDF + River + Road),θ(.),θ'(.),*p*(River) | 8 | 337 | 116.76 | 1.53 | 0.24 | 0.76 | 0.47 | 0.246 |
| ψ(PDF + River + Road),θ(.),θ'(.),*p*(River + Disturbance) | 9 | 337 | 118.26 | 3.03 | 0.11 | 0.87 | 0.22 | 0.248 |
| ψ(PDF + River + Road),θ(.),θ'(.),*p*(NDVI) | 8 | 337 | 118.53 | 3.30 | 0.10 | 0.97 | 0.19 | 0.067 |
| ψ(PDF + River + Road),θ(.),θ'(.),*p*(.) | 7 | 337 | 121.15 | 5.92 | 0.03 | 1.00 | 0.05 | 0.043 |
