# Supplementary material for: Rhinos in the Parks: An Island-Wide Survey of the Last Wild Population of the Sumatran Rhinoceros
Source: PLoS One. 2015 Sep 16;10(9):e0136643. doi: 10.1371/journal.pone.0136643 (PMC4574046; doi:10.1371/journal.pone.0136643)
Supplement: S5 Table — Model selection results; roles of covariates in determining probability of occupancy Sumatran rhino, with constant detection probability p on 1km long replicates, using the Hines et al. (2010) model. Number of sites = 28. Covariates considered Road Density (Road), Forest, Deforestation, and Curvature of NDVI (NDVI). (DOCX) [file pone.0136643.s012.docx]

### S5 Table. Way Kambas NP - 2008. Model selection results; roles of covariates in determining probability of occupancy Sumatran rhino*,* with constant detection probability *p* on 1km long replicates, using the Hines et al. (2010) model. Number of sites = 28. Covariates considered Road Density (Road), Forest, Deforestation, and Curvature of NDVI (NDVI).

| Model | Number of parameters | n | AICc | ΔAICc | AIC weight | Cumulative Weight | Model Likelihood |
| --- | --- | --- | --- | --- | --- | --- | --- |
| ψ(Road),θ(.),θ'(.),*p*(.) | 5 | 28 | 72.75 | 0.00 | 0.51 | 0.51 | 1.00 |
| ψ(Forest),θ(.),θ'(.),*p*(.) | 5 | 28 | 74.92 | 2.17 | 0.17 | 0.68 | 0.34 |
| ψ(Road + Forest),θ(.),θ'(.),*p*(.) | 6 | 28 | 75.56 | 2.81 | 0.12 | 0.80 | 0.25 |
| ψ(Road + NDVI),θ(.),θ'(.),*p*(.) | 6 | 28 | 75.99 | 3.24 | 0.10 | 0.91 | 0.20 |
| ψ(River + Deforestation),θ(.),θ'(.),*p*(.) | 6 | 28 | 76.11 | 3.36 | 0.09 | 1.00 | 0.19 |
