# Supplementary material for: Rhinos in the Parks: An Island-Wide Survey of the Last Wild Population of the Sumatran Rhinoceros
Source: PLoS One. 2015 Sep 16;10(9):e0136643. doi: 10.1371/journal.pone.0136643 (PMC4574046; doi:10.1371/journal.pone.0136643)
Supplement: S6 Table — Model selection results; roles of covariates in Sumatran rhinoceros occupancy in Leuser Landscape, based on modeling probability of detecting rhino sign p on 1km long replicates using the Hines et al. (2010) model. Number of sites = 28. Covariates considered Road Density (Road), Forest, Disturbance, Curvature of NDVI (NDVI), and River. (DOCX) [file pone.0136643.s013.docx]

S6 Table. Way Kambas NP – 2008. Model selection results; roles of covariates in Sumatran rhinoceros occupancy in Leuser Landscape, based on modeling probability of detecting rhino sign *p* on 1km long replicates using the Hines et al. (2010) model. Number of sites = 28. Covariates considered Road Density (Road), Forest, Disturbance, Curvature of NDVI (NDVI), and River.

| Model | Number of parameters | n | AICc | ΔAICc | AIC weight | Cumulative Weight | Model Likelihood | Cond Psi total average by area |
| --- | --- | --- | --- | --- | --- | --- | --- | --- |
| ψ(Road),θ(.),θ'(.),p(.) | 5 | 28 | 72.75 | 0.00 | 0.51 | 0.51 | 1.00 | 0.448 |
| ψ(Road),θ(.),θ'(.),p(Forest) | 5 | 28 | 74.85 | 2.10 | 0.18 | 0.69 | 0.35 | 0.548 |
| ψ(Road),θ(.),θ'(.),p(Disturbance) | 6 | 28 | 75.88 | 3.13 | 0.11 | 0.80 | 0.21 | 0.462 |
| ψ(Road),θ(.),θ'(.),p(NDVI) | 6 | 28 | 75.91 | 3.16 | 0.11 | 0.90 | 0.21 | 0.453 |
| ψ(Road),θ(.),θ'(.),p(River) | 6 | 28 | 76.02 | 3.27 | 0.10 | 1.00 | 0.20 | 0.448 |
