# Supplementary material for: Rhinos in the Parks: An Island-Wide Survey of the Last Wild Population of the Sumatran Rhinoceros
Source: PLoS One. 2015 Sep 16;10(9):e0136643. doi: 10.1371/journal.pone.0136643 (PMC4574046; doi:10.1371/journal.pone.0136643)
Supplement: S7 Table — Model selection results; roles of covariates in determining probability of occupancy Sumatran rhino, with constant detection probability p on 1km long replicates, using the Hines et al. (2010) model. Number of sites = 55. Covariates considered Primary Dryland Forest (PDF), River, Secondary Dryland Forest (SDF), Deforestation, and Disturbance. (DOCX) [file pone.0136643.s014.docx]

### S7 Table. Bukit Barisan Selatan NP – 2010-2011. Model selection results; roles of covariates in determining probability of occupancy Sumatran rhino*,* with constant detection probability *p* on 1km long replicates, using the Hines et al. (2010) model. Number of sites = 55. Covariates considered Primary Dryland Forest (PDF), River, Secondary Dryland Forest (SDF), Deforestation, and Disturbance.

| Model | Number of parameters | n | AICc | ΔAICc | AIC weight | Cumulative Weight | Model Likelihood |
| --- | --- | --- | --- | --- | --- | --- | --- |
| ψ(PDF + River),θ(.),θ'(.),*p*(.) | 6 | 55 | 282.16 | 0.00 | 0.43 | 0.43 | 1.00 |
| ψ(SDF + River + Deforestation),θ(.),θ'(.),p(.) | 8 | 55 | 283.39 | 1.23 | 0.23 | 0.67 | 0.54 |
| ψ(PDF + River + Disturbance),θ(.),θ'(.),*p*(.) | 7 | 55 | 283.70 | 1.54 | 0.20 | 0.87 | 0.46 |
| ψ(PDF + Disturbance),θ(.),θ'(.),p(.) | 6 | 55 | 285.88 | 3.72 | 0.07 | 0.94 | 0.16 |
| ψ(PDF),θ(.),θ'(.),*p*(.) | 5 | 55 | 286.05 | 3.89 | 0.06 | 1.00 | 0.14 |
