# Supplementary material for: Rhinos in the Parks: An Island-Wide Survey of the Last Wild Population of the Sumatran Rhinoceros
Source: PLoS One. 2015 Sep 16;10(9):e0136643. doi: 10.1371/journal.pone.0136643 (PMC4574046; doi:10.1371/journal.pone.0136643)
Supplement: S8 Table — Model selection results; roles of covariates in Sumatran rhinoceros occupancy in Leuser Landscape, based on modeling probability of detecting rhino sign p on 1km long replicates using the Hines et al. (2010) model. Number of sites = 55. Covariates considered Primary Dryland Forest (PDF), River, Dryland Agriculture (DLA), Disturbance, and Forest. (DOCX) [file pone.0136643.s015.docx]

### S8 Table. Bukit Barisan Selatan NP – 2010-2011. Model selection results; roles of covariates in Sumatran rhinoceros occupancy in Leuser Landscape, based on modeling probability of detecting rhino sign *p* on 1km long replicates using the Hines et al. (2010) model. Number of sites = 55. Covariates considered Primary Dryland Forest (PDF), River, Dryland Agriculture (DLA), Disturbance, and Forest.

| Model | Number of parameters | n | AIC | ΔAIC | AIC weight | Cumulative Weight | Model Likelihood | Cond Psi total average by area |
| --- | --- | --- | --- | --- | --- | --- | --- | --- |
| ψ(PDF + River),θ(.),θ'(.),*p*(DLA) | 7 | 55 | 277.08 | 0.00 | 0.54 | 0.54 | 1.00 | 0.330 |
| ψ(PDF + River),θ(.),θ'(.),*p*(DLA + Disturbance) | 8 | 55 | 278.94 | 1.86 | 0.21 | 0.75 | 0.39 | 0.330 |
| ψ(PDF + River),θ(.),θ'(.),*p*(DLA + Disturbance + Forest) | 9 | 55 | 280.35 | 3.27 | 0.10 | 0.85 | 0.20 | 0.320 |
| ψ(PDF + River),θ(.),θ'(.),*p*(Forest) | 7 | 55 | 281.86 | 4.78 | 0.05 | 0.90 | 0.09 | 0.340 |
| ψ(PDF + River),θ(.),θ'(.),*p*(.) | 6 | 55 | 282.16 | 5.08 | 0.04 | 0.94 | 0.08 | 0.340 |
| ψ(PDF + River),θ(.),θ'(.),*p*(Disturbance + Forest) | 8 | 55 | 282.56 | 5.48 | 0.03 | 0.98 | 0.06 | 0.310 |
| ψ(PDF + River),θ(.),θ'(.),*p*(Disturbance) | 7 | 55 | 283.51 | 6.43 | 0.02 | 1.00 | 0.04 | 0.343 |
